# The development of large-scale de-identified biomedical databases in the age of genomics—principles and challenges

**DOI:** 10.1186/s40246-018-0147-5

**Published:** 2018-04-10

**Authors:** Fida K. Dankar, Andrey Ptitsyn, Samar K. Dankar

**Affiliations:** 1 College of IT, UAEU, Al Ain, UAE; 2 Gloucester Marine Genomics Institute, Gloucester, MA USA; 3 Faculty of Sciences, University of Balamand, Souk El Ghareb, Lebanon

**Keywords:** Biomedical database, Data privacy, Data governance, Whole genome sequencing

## Abstract

Contemporary biomedical databases include a wide range of information types from various observational and instrumental sources. Among the most important features that unite biomedical databases across the field are high volume of information and high potential to cause damage through data corruption, loss of performance, and loss of patient privacy. Thus, issues of data governance and privacy protection are essential for the construction of data depositories for biomedical research and healthcare. In this paper, we discuss various challenges of data governance in the context of population genome projects. The various challenges along with best practices and current research efforts are discussed through the steps of data collection, storage, sharing, analysis, and knowledge dissemination.

## Background

### Overview

Databases are both the result and the instrument of research. From the earliest times, assembling collections of samples and stories was essential for any research project. The results of research feeding back into the libraries and collections create a positive feedback in the accumulation of knowledge limited only by the technological platform for storage and retrieval of information. The modern times did not change the principle but further emphasized it with the advent of computers, mass information storage, and high-throughput research instrumentation. Modern biomedical databases may vary in size, specialization, and type of access but with a few exceptions are voluminous and include complex data from multiple sources. Arguably, the first integrated database of the population scale was initiated in Iceland when Decode Genetics started in 1996 [1]. This new generation of integrated biomedical databases incorporates both phenotype (medical records, clinical studies, etc.) and genotype (variation screening at first, now increasingly shifting to whole exome and whole genome sequencing [2, 3]). The project started by Decode has generated one of the best resources for discovery in biomedical sciences and inspired development of multiple populational and national genomics projects, also feeding into integrated databases. Genomics England [4], Human Longevity [5], All of US (formerly known as Precision Medicine Initiative) [6], China’s Precision Medicine Initiative [7], Korean Reference Genome Project [8], Saudi Human Genome Program [9], and Qatar Genome [10] programs are just a few recent examples of active large-scale projects generating enormous databases of complex biomedical information. Large-scale population genomics projects proliferating in the second decade of the twenty-first century show enormous diversity in goals and strategies. The Icelandic genome program has evolved from the largest population genetics study of the time and has primary objectives in advancing biomedical research. China’s Precision Medicine Initiative is one of the most ambitious programs with an aim to sequence 100 million whole human genomes by 2030. The objective is to improve disease diagnosis, develop targeted treatments, and provide better wellness regimes. Genomics England is an augmented (100,000) research cohort study that implies sampling of the most common diseases and reflecting the genetic diversity of the population in Great Britain. The All of Us project has similar objectives and aims to collect a sufficiently large cohort (1,000,000). The numbers alone have a great ameliorating effect on statistical power of association studies. Deep phenotyping and follow-up sampling in All of Us are aiming to develop the new level of precision in diagnostic and treatment of multiple diseases. The declared aims of the Human Longevity project are even more focused on a specific range of age-associated diseases. To achieve its goals, Human Longevity plans to recruit about 1,000,000 donors. The Saudi Human Genome Program has a very different focus; it aims to develop effective methods and facilities for early diagnostics and treatment of heritable diseases. Such goal does not require the genome sequencing effort on the same scale as All of Us or Genomics England. The program implements only a small number of whole genome sequencing and up to 100,000 whole exome sequencing to collect the data reflecting local genetic variation and design a microarray chip for cost-effective mass neonatal screening. In contrast, the national genome program in Kuwait requires complete sampling of the entire population including nationals and non-citizen residents because the principal goal, according to the recently adopted DNA Law [11], is to counteract terrorist activity by precise unequivocal identification of every human being. The Qatar Genome Programme (QGP) aims to integrate genome sequencing information of all Qatari nationals with electronic medical records (EMRs) and results of clinical studies to provide quick and precise personalized diagnostic and treatment of diseases. The goal is to provide a solid basis for the biomedical research in the country.

These biomedical databases are often viewed as a platform for regional and worldwide collaborative research projects. Both the construction of these resources and serving them to a growing research community (national and international) present a significant challenge toward preserving the privacy of the participants.

### Particularities of genomic data

In 2008, James Watson, a co-discoverer of the double-helix DNA model, opted to release his sequenced genome in a public database with the exception of his APOE gene (which has been associated with Alzheimer’s disease). However, a statistical model was later developed that inferred the missing gene with a high degree of confidence [12]. This incident conveys one of many new privacy concerns that genomic data raises and that are difficult to deal with:

- First, genomic data is highly distinguishable. There is confirmation that a sequence of 30 to 80 SNPs could uniquely identify an individual [13]. Genomic data is also very stable [14]. It undergoes little changes over the lifetime of an individual and thus has a long-lived value (as opposed to other biomedical data such as blood tests which have expiry dates).
- Second, genetic data provides sensitive information about genetic conditions and predispositions to certain diseases such as cancer, Alzheimer, and schizophrenia. If breached, such information can be stigmatizing to participants and can be used against them in employment and insurance opportunities, even if these pre-dispositions never materialize.
- Third, genetic data does not only provide information about the sequenced individuals but also about their ancestors and off springs. Whole genome data increases our ability to predict information related to relatives’ present and future health risks, which raises the question as to the obligation of a consented participant towards their family members (the authors in [15] describe privacy risks to family members of individuals who shared their genetic data for medical research).
- Finally, and most concerning, there is great fear from the potential information hidden within genomic data [16]. As our knowledge in genomics evolves, so will our view on the sensitivity of genomic data (in other words, it is not possible to quantify the amount and sensitivity of personal information that can be derived from it).

### Paper outline

In this paper, we discuss various privacy and governance challenges encountered during the construction and deployment of population-scale sequencing projects. The various challenges are discussed through the stages of:

1. Initial data collection,
2. Data storage,
3. Data sharing (utilization), and
4. Dissemination of research findings to the community.

At each stage, we discuss current practices and challenges, as well as contemporary research efforts, with a particular interest in *data sharing for research purposes* [17]. We provide examples from a diversity of large-scale population sequencing projects and reflect on their scope and data governance models.

Note that the above division is simplistic as the different stages are not mutually exclusive; however, it makes for a simpler and more organized presentation of the different ideas.

## Data collection

The data for the different genome projects is sought from the community and results from the efforts on part of the community. Thus, it is important to consult with the concerned population to establish the basic principles for data collection and research oversight. To achieve that, *a community engagement model* should be defined. The model should establish the basic principles for data collection and research oversight such as:

(i) *An advocating technique* for advertising the project to the community and raising the number of individuals who are aware of the project. Such technique should strive to reach different elements within the society, provide clear dissemination of risks and benefits, and establish methods for recurrent evaluation of the community attitudes and understanding of the project.
(ii) *Enrollment criteria* to define the basis for enrollment (should it be disease-based or volunteer-based) as well as the acceptable age for volunteers.
(iii) *An enrollment process* to define the scope of subjects’ consent (a general opt in/out or an informed consent) and to set a clear boundary between research and clinical practice, and
(iv) *An institutional and community-based oversight process* to discuss and establish oversight for the program by the community and by independent ethics committees. The scope of these committees should include oversight on data repositories, oversight on research studies and oversight on any changes to the protocol (data use agreements, communications, etc.).

In many cases, regulations require the organization to establish an independent institutional review board, (IRB). The IRB’s mandate (at the data collection and storage phases) is to review and approve all proposals related to the data collection protocol and to approve/manage the participant’s consent process for the data collection activity.

One of the most comprehensive community engagement models is that of the Electronic Medical Records and Genomics (eMERGE) network [18]. eMERGE, a National Institute of Health Initiative, is a consortium of nine US medical research institutes (including Vanderbilt Genome-Electronic Records (VGER) project and North Western University biorepository (NUgene)) that combine DNA repositories and EMR systems for advancing genetic research. In the case of VGER [19], the community engagement model was established in consultation with the community through surveys, focus groups (from different ethnic, racial, and socioeconomic backgrounds), posters, and in-person interviews. These activities helped in shaping the principles of data collection, data sharing, and community oversight. The established oversight bodies include The Vanderbilt IRB, the medical center’s ethics committee, and several newly established ethics, scientific, and community advisory boards. The community advisory board’s role is to evaluate the projects’ adherence to the established security and privacy measures, to voice the concerns/issues of the community with regards to the use of their genetic information for research, and to monitor any social/ethical issues arising in the process and help in providing the necessary measures to resolve them [19].

In the case of the NUgene project (North Western University biorepository, another eMERGE network member), the NUMC (Northwestern Medical Center) scientific, medical, and ethics community; the North Western University IRB; community researchers; external advisors; and public health experts were all involved early in establishing issues of consent for genome-wide association studies (GWASs), means to inform participants about data sharing, means to keep participants informed about research activities, and means to engage participants and learn their concern regarding data sharing.

For the case of the Qatar Genome Programme, oversight is provided mainly by an IRB and an access committee (involving prominent members of the community). Although some effort was exercised to publicize the long-term goals and benefits of the project and to get the community involved, the major recruitment incentive is the comprehensive health check provided as part of the sample collection visits by the Qatar Biobank [10]. The appointment takes two 2 days and includes an extensive set of studies and measurement. The measurements include height, weight, blood pressure, grip strength, waist and hip measurements, and body fat composition. The study proceeds to lung function, ultrasound carotid artery scan, 12-lead electrocardiogram, full body iDXA scan, artery stiffness measurement, and treadmill walking test. Finally, samples of blood, saliva, and urine are collected and analyzed.

Most large-scale population genomics programs collect some phenotypic data; the type and volume adjusted to the goals of the study. For instance, the Estonian Genome Project data collection is performed by the Estonian Biobank. The emphasis is on collection of personal data by computer-assisted personal interview (CAPI) within hours of appointment at a doctor’s office. The CAPI includes personal and genealogical data (place of birth, ethnicity, family history of medical conditions, etc.), educational and occupational history, and lifestyle data (physical activity, dietary habits, smoking, alcohol consumption, etc.). During the appointment, additional anthropometric, blood pressure, and heart rate data are collected along with the blood sample. The particular feature of the Estonian Genome Project is its strong association with electronic health records providing access to the past and current health status of each sample donor. However, the phenotype study is by far less intensive than that of the Qatar Genome Programme. Saudi Human Genome Program [20] collects virtually no individual phenotype data since this information is not essential to the goals of the program. In the most extreme example, the Kuwait DNA Law [11] showed no interest in phenotype data; mandatory DNA sampling from all residents and visitors also implied no need for consent on the part of the sample donor. Remarkably, after the international outcries pointing out potential abuse of such law, local protests, and challenge from the lawyers, the law has been amended in its most controversial parts.

Protecting participants’ data from privacy breaches is a key issue to the success of any genome project. Prospective participants in research studies ranked privacy as one of their top worries and as a major determinant toward their participation in a study [21–23]. Privacy is a socially bound concept; it is deeply affected by language, religion, traditions, and cultural expectations. A simple question such as “how much rent do you pay?” is considered inappropriate in some societies while perfectly normal in others. In the Arab world, for example, personal reputation and family ties are among the highest moral values. As explained by Abokhodair and Vieweg [24], “membership in a family or tribe are of the utmost importance; there is no individual separate from a family … asserting one’s individuality is viewed in a negative light”; in fact, individuals often rely on their family members and communities for significant decisions, while in western societies, asserting one’s individuality is celebrated. For these reasons, privacy breaches from genetic testing may differ in their impact on individuals from different backgrounds. Thus, it is important to investigate and understand the cultural values of concerned communities and to tailor the specifics of data collection and data sharing accordingly. Unfortunately, privacy is still treated as a universal notion, and little research has been done to understand the cultural impact.

In the next two sections, we discuss current practice and challenges in protecting participants’ sensitive data while in storage (data storage) and while in use (data sharing).

## Data storage

EMR and Biobank data are highly sensitive and require significant storage space (the total length of an individual genome is over 3 billion base pairs). As such, one of the biggest challenges for a data warehouse is to decide *where* and *how* to store this data.

### Where to store the data?

Data storage presents a significant technological challenge for many large-scale genome projects. The total volume of deep whole genome sequencing (WGS) with raw read, aligned, and variant calling data can reach 0.5 TB per genome. Phenotyping, imaging, and omics data add additional volume. The specific number may vary widely depending on the types of data collected. Questionnaires and physiological tests, even as comprehensive as those conducted by Qatar Biobank, when collecting samples for the Genome Sequencing Program, add only a small percent to the total volume. Digital images can potentially add large volumes on the same scale as genome sequencing (i.e., on TB scale). However, the real imaging data associated with a particular sample donor in current projects is relatively small and does not exceed gigabyte (GB) scale. Omics data (such as gene expression, methylation, or metabolomics) can also be as large as genome sequencing data. Some of such data is produced using similar next-generation sequencing techniques that result in the same volumes of raw data, which can be stored to reproduce the downstream analysis. Multiple tissue samples can be taken for omics analysis from different organs of the same donor, at different times or in different disease states. This potentially can multiply the volume of data by as many times as more samples are taken. However, at this time, this kind of data is rarely added in significant amounts due to the high costs of high-throughput methods. WGS data remains the most voluminous part of genomic databases. With reserve copy and redundancy, the overall data volume requires petabytes of storage space even for relatively small population studies with tens of thousands of samples. Data compression and selective saving of key data files (while other types of data can be reproduced from initial and intermediate data) can reduce the requirements. Nevertheless, the overall data storage demand in population sequencing is enormous. In the QGP example, it has been originally estimated as 300 PB. The challenge is further compounded by requirement of fast access to individual data files, high-throughput access to multiple genomes in research cohort studies, and long-term storage keeping the data safe and actively used for decades ahead. On the other hand, the price of storage has a hard ceiling dictated by the progress in sequencing technology: the price for data storage per gigabyte should not exceed (and better be significantly lower than) the price of sequencing of the same data from a stored sample. Such demands and limitations make engineering the data storage facility extremely challenging.

**Fig. 1:**
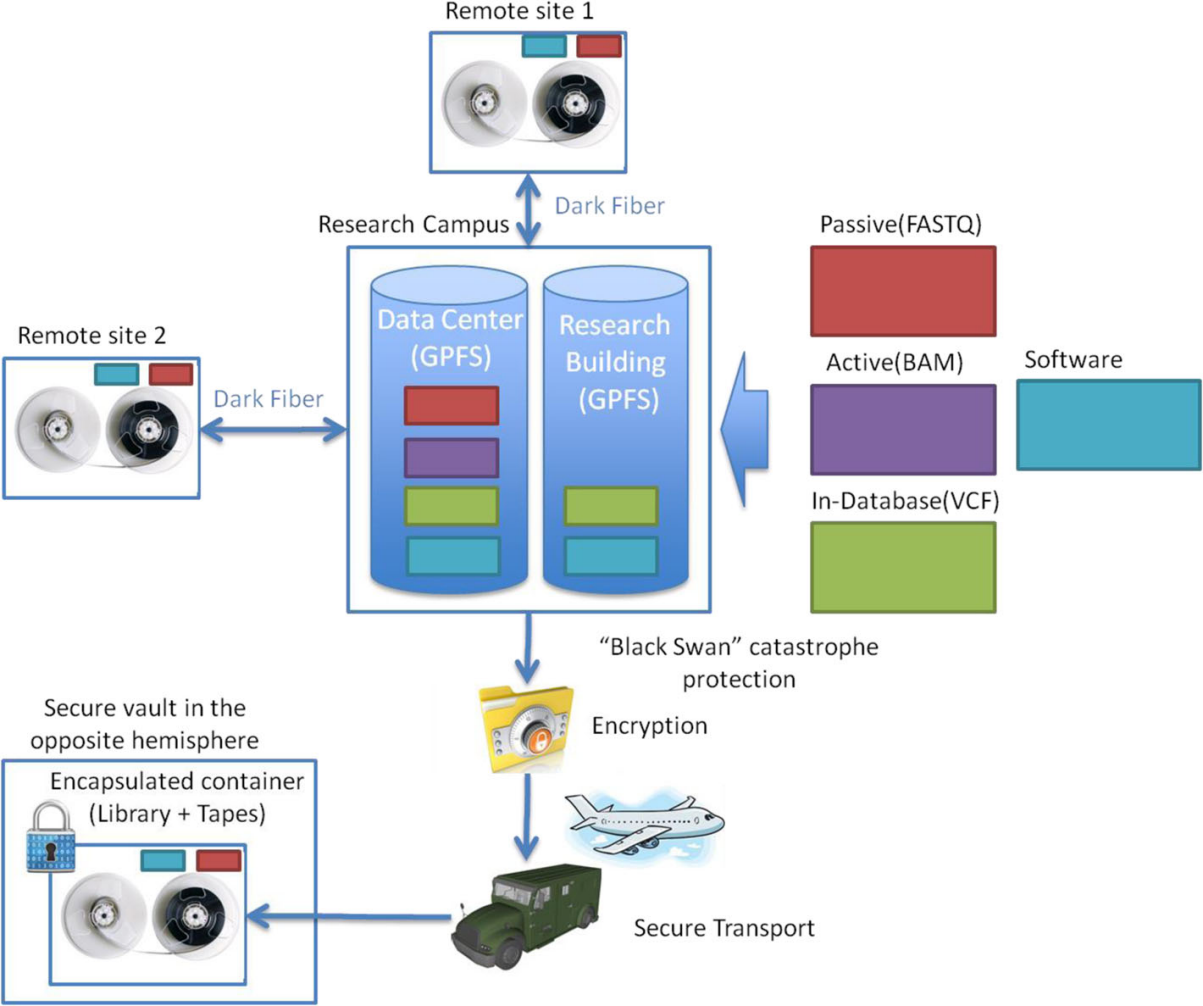
Secure storage strategy for a large-scale population sequencing project. All data is stored in a secure data center with partial mirroring for research on site, partial archival mirroring for backup at geographically distant remote sites within the country, and additional mirror copy for protection against unforeseeable rare catastrophic (aka “Black Swan”) events.

In general, the data can be outsourced to a cloud provider or stored on a private—locally managed—cloud. The former approach obscures the complexity of technology but demands highly developed broadband network infrastructure and limits the control over data security and access performance. The overall performance of a cloud-based data storage solution in a large-scale project is gated by the availability of broadband infrastructure. Nevertheless, when local conditions offer adequate answers to security and broadband infrastructure challenges, cloud solution can be very attractive. Genomics England with a goal of 100,000 WGS and full complement of phenotype data is the most brilliant example [25]. The latter approach can be more expensive in terms of engineering, capital expenses, and running costs. In the QGP example, the storage is engineered as a complex solution that involves multiple redundancy and multi-tier storage on different information carriers ranging from flash drives to tape libraries. However, the storage service is provided in a form of a single name space private cloud (see overview in Fig. 1).

In other examples of local storage solution for large-scale genomic and biomedical data, the technical details of storage architecture are rarely detailed and rely on the local policies of the data center for data integrity, security, and safety. Examples of such projects include the Estonian Genome Project and Saudi Human Genome Program [26, 27].

It is increasingly advocated that individuals should be the guardians of their own biomedical data. As such, they should have the ability to access, modify, and grant access (to family, health authorities, or research facilities) as they see fit. However, numerous challenges (in terms of data storage) have to be solved before such model can be adopted, such as:

1. Where should individual data be stored (individual’s private PC or on a private access-controlled cloud?), and how to ensure the security of the data in either case?
2. How to grant access to different authorities and how to manage such access?
3. Should the data be backed up, where and how?
4. Does the individual have the right to withdraw authorized access or to delete their data, and how can either be done [28]?

### How to store the data?

**Fig. 2:**
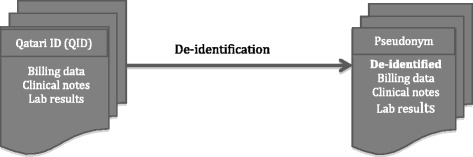
De-identification of clinical data

To minimize the risk of harm, most research platforms store de-identified clinical and biobank data while retaining the link between both data sources (the de-identified EMR data and the biobank data). This can be achieved by applying the following two operations:

1. The first operation (known as pseudonymization) identifies a stable and unique identifier(s) (such as Social Security numbers and national IDs) that is included in both data sources and replaces it with a unique random ID or pseudonym (refer to Fig. 2). The pseudonym can be obtained by encrypting or hashing one or several identifiers. Decode genetics uses a symmetric encryption algorithm (TwoFish) to convert the Social Security number (SSN) to an alphabet-derived string. VGER hashes the medical record number using the public hashing algorithm SHA-512.
2. The second operation removes all uniquely identifying information (such as names, record number, and emails) from the structured data and masks all unique identifiers from the unstructured data (such as doctors’ notes), (refer to Table 1 for examples of unique identifiers). Additional fields can be also removed from the data for added privacy; the VGER project, as an example, removes all geographic information smaller than a state and all elements of dates (except year) directly related to the individual (such as date of birth and date of death) and shifts all hospital visit dates by a random value between 1 and 364 days (the shift being the same across the record of the same patient to preserve temporal analysis).

**Table 1:** Examples of unique identifiers

Uniquely identifying fields	Remarks
National ID (or SSN)	
Name	Names of patients and caregivers
Email	
Source ID	Hospital/Biobank-assigned IDs
Passport number	
Exact address	

Multiple aspects have to be considered when designing the pseudonymization operation; these include:

1. Ensuring that each subject is assigned the same random ID (pseudonym) across different data sources. This consistency will ensure that data belonging to a particular subject will always be mapped to one record.
2. Deciding whether the pseudonymization process should be reversible or not. Reversible systems allow reverting back to the identity of the subjects through a process called de-pseudonymization. For the case of Decode Genetics and QGP, reversibility was chosen because communication with patients was deemed to be a foreseen possibility (to communicate novel treatments and/or possible preventative measures). While for the VGER case, reversibility is not possible as the link between the pseudonym and the medical record number was not maintained.
3. When communication is forecasted, a secure de-pseudonymization mechanism should be specified; the mechanism should define (i) the cases for which de-identification can occur, (ii) the bodies that can initiate re-identification requests, (iii) those that rule and regulate these requests, and (iv) the actual re-identification mechanism.

Privacy breaches can occur if the data is leaked to an unauthorized party. Such leakage can happen if (i) the *stored data is hacked/handled recklessly* or if it is (ii) *shared with a pretentious/irresponsible third party*. After applying the pseudonymization process, the data remains vulnerable to de-identification attacks (in other words, although de-identification makes re-identification harder, it does not eliminate the risk). Thus, a strong security layer is needed to ensure that unauthorized individuals cannot access/modify the data. Encryption alone is not an adequate security solution, particularly for genomic data. As explained in [28], encryption schemes gradually weaken in the long run, while the information hidden inside a genome remains stable and is better interpreted with time. Thus, if encrypted genomes are available to an unauthorized third party, the party will be able to decrypt it with time (40–50 years).

Commercial cloud providers (such as IBM and Amazon) claim to employ foolproof security, but their models are not shared publicly and thus cannot be learned and evaluated. Security of the privately held infrastructure and private clouds depends on the proficiency of system administrators and security specialists employed by the custodian organization. In some cases, like the Qatar Genome Programme, geographic location and state-regulated data access may provide additional protective layer against sporadic attacks and “social engineering” hacks. However, the ability of genomic data storage to withstand a determined and competent invasion is yet to be tested.

## Data sharing

Electronic medical records (EMRs) hold diverse clinical information about large populations. When this information is coupled with genetic data, it has the potential to make unprecedented associations between genes and diseases. The incorporation of these discoveries into healthcare practice offers the hope to improve healthcare through personalized treatments. However, the availability of such data for widespread research activities is dependent *on the protection of a subject’s privacy*. Current technological methods for privacy preservation are outdated and cannot provide protection for genomic and longitudinal data (EMR).

### Access mechanisms and privacy

Data sharing mechanisms can be categorized into two broad categories: open-access and controlled-access. While both were widely used for regulating genomic data sharing, open-access datasets have been used in many more studies per year [29]. Open-access models either operate under a mandate from participants (who want to publish their genomic data in public platforms) or under the assumption that the shared data is de-identified and possibly aggregated [30]. However, as demonstrated by multiple recent studies, the risk of re-identification is strongly present. It was shown, in multiple independent studies, that it is possible to learn the identities of people who participate in research studies by matching their data with publicly available data [31]. In a recent study [32], the authors showed that they can infer the identity of 50 anonymous male subjects whose Y-chromosome has been sequenced as part of the 1000 Genomes Project. The researchers were not only able to discover the identities of these anonymized research participants but also their family members using available/public pedigrees. In response to this study, the NIH removed the age information from the project’s database. In another recent study, [33, 34], the authors reported that they can confirm whether a person participated in a genome-wide association study, by using information from the person’s DNA sample, “even if the study reported only summary statistics on hundreds or thousands of participants” [31]. In response, the NIH shifted to a controlled access mechanism. In fact, currently, most human genome projects use controlled-access mechanisms.

The personal information derived from genomic data (and EMR data) can be very damaging to the participants. It can be used against them to limit insurance coverage, to guide employment decisions, or to apply social stigma. In [35], the authors report on a case of genetic discrimination by a railroad company. The case occurred in 2002 when the company forced its employees to undergo a genetic test; employees who refused to participate in the test were threatened with disciplinary actions. The company was later forced (in an out-of-court settlement) to compensate 36 of its employees. That is hardly a consolation because if such genetic data was obtained from online sources or breached through illegal means, the company may have been able to get away with its discrimination practices.

### Regulations

In many countries, the use of sensitive human-subject data for research purposes has been studied extensively from the legal aspect. Resulting legislations aimed to ensure that private information is properly used and adequately protected when disclosed for research purposes [36, 37]. The legislations (such as the Common Rule [36], Health Information Portability and Accountability Act (HIPAA) [38], and EU data protection directive [39]) generally permit data sharing under one of the following guidelines:

1. For the use of identifiable data, an approval from an Institutional Review Board (IRB) is required. To approve data requests, IRBs require:
  - Informed consents from the participants for the specific data use, or
  - When consents are deemed impractical, IRBs can grant data access if the study accrues more benefit than risk. Such decision requires a thorough and lengthy evaluation of each data access request from the IRB part.
2. For adequately de-identified data, researchers can be exempt from IRB approval. The adequacy of the de-identification is generally established by the IRB or by pre-approved policies such as the United States HIPAA privacy rule [37].

Guideline G2 depends on the availability of robust de-identification techniques, but as current techniques are outdated, and unable to deal with genetic and EMR data (as evident from the privacy breaches cited earlier), G2 cannot be adopted. The Vanderbilt genome project is the only project we are aware of that was ruled by Vanderbilt IRB to be a “non-human subject data” as it was deemed to be properly de-identified. However, given the potential impact of the project on the community, guidelines adhering to G1.b were enforced.

Guideline G1.a requires informed consent from participants. The problem with such requirement is that data collectors have to forecast all possible uses of the data and create a comprehensive consent detailing the benefits and risks related to all different data uses. Something that is not easily achievable. In fact, most biobanks collect consents in the form of opt in/opt out [19]. The issues/challenges in implementing proper informed consent will be discussed in depth later in this section.

Almost all existing biomedical data warehouses that house (non-aggregate) genetic data coupled with EMR data follow guideline *G1.b*. These warehouses lightly de-identify their data and regulate investigators’ access to the data through an IRB [18, 19, 40]. Only researchers with studies that involve less risk than benefit are allowed access to requested data and only after they pass a thorough identity check. However, IRB procedures are extensive and can obstruct timely research and discoveries [41–43]. Studies on platforms that rely on IRB for all data accesses reveal unsatisfied users. The application process is strenuous and approvals take a long time often delaying project initiation significantly [43, 44].

In Qatar, as an example, access to the biomedical data collected in Qatar is governed by the QSCH “guidelines, regulations and policies for research involving human subjects”, which adheres to guideline *G1.b*. A recently formed IRB will regulate all accesses to the research data and services by all research institutes within Qatar and outside.

With such massive mandates, a principal feature for IRBs is to have the capacity to foster timely research and discoveries. Data application processes and approvals should be smooth and should not delay project initiation significantly. Thus, the traditional “IRB-based” data sharing will produce unsatisfied users.

### Methods under investigation

The inadequacy of current de-identification methods and the delays in IRB processes prompted privacy experts to seek new solutions. Rapid progress is taking place in privacy research in the biomedical area, driven by the need to protect and benefit from the large biomedical data warehouses being built worldwide. The novel methods can be divided into two main categories, legislative and technical:

(i) *Legislative:* Legislative methods define privacy rights and responsibilities. Research in this area aims to understand and define individuals’ privacy perspectives and expectations and to update policies and laws that govern data sharing. Genetic data introduces a difficult and unique regulatory situation (with respect to data collection laws and data sharing laws) that is not found with other types of health data [16]. So, until effective privacy protection solutions are made into law, scientists and civil right advocates are calling for the adoption of anti-genetic discrimination laws to mitigate the effect of genetic data breaches. An example is the Genetic Information Non-discrimination Act (GINA) adopted by the US government in 2008. GINA forbids discrimination by insurers or employers on the basis of genetic information. The problem with such regulations is that they are enforced only when discrimination on the basis of genetic information is proven, which necessitates the difficult task of proving malicious intentions.
(ii) *Technical:* Technical controls aim to create data sharing systems/methods that fulfill the requirements specified in privacy legislation. Current technical approaches to privacy, such as de-identification, are not effective in the genomic context (in fact, the genome is itself an identifier and as such cannot be de-identified (yet) while retaining its utility), thus the need for innovative methods to deal with our new data realities. We classify current research in privacy-preserving mechanisms into three categories: process-driven mechanisms, risk-aware systems, and consent-based systems. In process-driven mechanisms, such as differential privacy and cryptographic techniques, the dataset is held by a trusted server, users query the data through the server, and privacy is built into the algorithms that access the data. Risk-aware systems aim at speeding the IRB processes through partial/full automation, and consent-based systems aim to empower participants by allowing them to control how and by whom their data can be used. This is being done through the introduction of novel dynamic consent mechanisms.

In what follows, we briefly describe recent efforts within each of the three technical categories.

#### Dynamic consent

Consent-based mechanisms provide data subjects with control over who can access their stored data/specimens, for what purposes, and for how long. Thus, a researcher requesting access to data will receive the data records for which the consent is fulfilled.

The current (mostly paper-based) consent process is static and locks consent information to a single time point (typically during sample collection) [45], requiring all future data usages to be specified at the time of initial consent. This is not feasible with current (multi-purpose and evolving) biomedical data warehouses. The current process also requires limiting the amount of information conveyed to participants to ensure that their consent is informed (i.e., the educational program), since individuals can only absorb limited information at any one time. Re-contacting participants to obtain additional consents and/or to provide additional education materials is arduous, time-consuming, and expensive. Moreover, it can have a negative impact on the participants and on the enterprise.

Active research is underway to overcome this problem. It attempts to provide consent dynamicity to make it easier on the participants and data holders to continuously provide/update consent information. The authors of [46] are working on ways to represent and manage consent information. They focus on defining the different dimensions of a consent. Such dimensions include (i) the characteristics of the institutions that can access the patient’s data, (ii) the level of details that each institution can access, and (ii) the type of research allowed on the data (all possible uses of the data). The authors’ approach is to codify the different consent dimensions. The benefit of the codification “is to provide a common language to capture consented uses of data and specimens” and to “select those data for the investigator’s study that are compliant with the subjects’ consented uses and the investigator’s permissions.” Thus, given a particular study, the characteristics of the study could be matched against the subjects’ codified consent to determine the data subset that conforms. In [47, 48], the authors discuss several challenges in designing dynamic consents, particularly, participant’s consent withdrawal and its implications. It is worth noting that some commercial sequencing companies, such as 23andme [49], already provide a limited form of dynamic consent models through secure online portal systems. Such systems allow users to fill/change their consent information at their own will.

Additional aspects that need to be resolved are consent withdrawal, continuous participant education, and the cultural aspect of the consent:

- *Consent withdrawal:* Withdrawal is an essential motivator for research participation; thus, research participants must be allowed to withdraw their participation at any time without any penalties. However, withdrawal is complicated by the fact that participants’ samples/data may already have been shared by other research organizations. Current best practices recommend that any leftover specimens be discarded and that medical data no longer be updated or used but that shared samples and data do not necessarily need to be revoked [50]. It is important for the consent process to highlight these issues and to make sure that participants understand the limitations of consent withdrawal. Additionally, more investigation should be done around different forms of withdrawals to understand their impact on the willingness to participate and to update best practices accordingly.
- *Continuous participants’ education:* Biomedical sciences are complex and are evolving very fast, which warrants the need for continuous participant education.
- *Cultural aspect:* The purpose of informed consent is to give the right of self-determination to individuals based on complete understanding of risks and benefits of research participation and without any interference or control by others. However, the right of self-determination is deeply affected by culture (some communities value the relationship with family members and turn to them for support when making critical decisions), and thus, consent should be adapted to the specifics of the underlying culture in terms of information sharing and disclosure [51].

#### Risk-aware access control

The risk of granting data access to a user depends on the characteristics of the request. For example, as stated in [52], “access to highly sensitive data at the data-holder’s location by a trusted user is inherently less risky than providing the same user with a copy of the dataset. Similarly, access to de-identified clinical data from a secure remote system is inherently less risky than access to identifiable data from an unknown location.” Risk-aware access control tries to quantify the risk posed by a data request and to apply mitigation measures on the data to counter the posed risk.

Risk aware access control received growing attention in the past few years. Several of the studies attempted to quantify/model privacy risk, both from the participants’ perspective and the data holder’s perspective. In [53], Adams attempts to model users’ perceptions of privacy in multimedia environments. He identified three factors that determine users’ perceptions of privacy: information sensitivity (user’s perception of the sensitivity of the released information), information receiver (the level of trust the user has in the information recipient(s)), and information usage (costs and benefits of the perceived usages). Lederer [54] uses Adams’ model as a framework for conceptualizing privacy in ubiquitous computing environments in addition to the Lessig model [55] for conceptualizing the influence of societal forces on the understanding of privacy. These efforts concentrate on privacy quantification from the participant perspective rather than the data holder.

Barker at al. [56] introduce a four-dimensional model for privacy: purpose (data uses), visibility (who will access the data), granularity (data specificity), and retention (time data is kept in storage). Barker et al.’s model was later used by Banerjee et al. [57] to quantify privacy violations. Along the same lines, and in multiple consecutive studies [58, 59], El Emam et al. defined three criteria that contribute to privacy risk; these are users’ motives, the sensitivity of the requested data, and the security controls employed by the data requestor. The authors state that, according to their long experience in private data sharing [58, 60–62], these are the main criteria used (informally) by data holders.

Recently, in [52, 63], the authors defined a conceptual risk-based access model for a biomedical data warehouse; the model defines the risk posed by data requests using four dimensions:

1. Data sensitivity, or the extent of privacy invasion that would result from inappropriate disclosure of the requested data,
2. Access purpose, or the usages for which the data was requested,
3. Location of the investigator’s institution, which is critical for checking the privacy legislation (if any) that applies at the data requester’s end and whether the same laws are enforceable, and
4. User risk, which measures:
  - The user’s institution ability to secure the data (the research institution to which the user is affiliated). This is evaluated by looking at the privacy practices followed/enforced within their headquarters and
  - The risk associated with the particular user/requestor; it is measured by tracking whether the user caused any past inconveniences.

Once calculated, the risk is fed into an access control decision module. The decision module imposes mitigation measures to counter the posed risk. The defined data-sharing mechanism would impose more mitigation measures on requests of higher sensitivity. The mitigations could manifest as reductions in the granularity of the data (de-identification) and/or as restrictions on when and how a user can access the data. The implementation of this model still requires significant efforts toward (i) assigning sensitivities to the different data attributes, (ii) assigning a score to institutions’ privacy and security practices (such as certifications), and (iii) creating universal user records for storing data breach information.

The issue of assigning sensitivity to data attributes is gaining more consideration. In [64], the authors define a method to detect privacy-sensitive DNA segments in an input stream. In [65], the authors present a privacy test to distinguish degrees of sensitivity within different attributes recognized as sensitive.

#### Secure multiparty computation

Secure multiparty computations (SMCs) are an attractive approach that allows a researcher to run a function on data owned by multiple parties (each holding a fraction of the data to be analyzed). The calculation is carried out on the overall dataset without any party having to reveal any of their own raw data. Such scenario can be particularly useful for cross-institutional studies (or even cross-countries studies) particularly when no site has enough data to conduct the study in question (for example, studies on rare diseases).

**Fig. 3:**
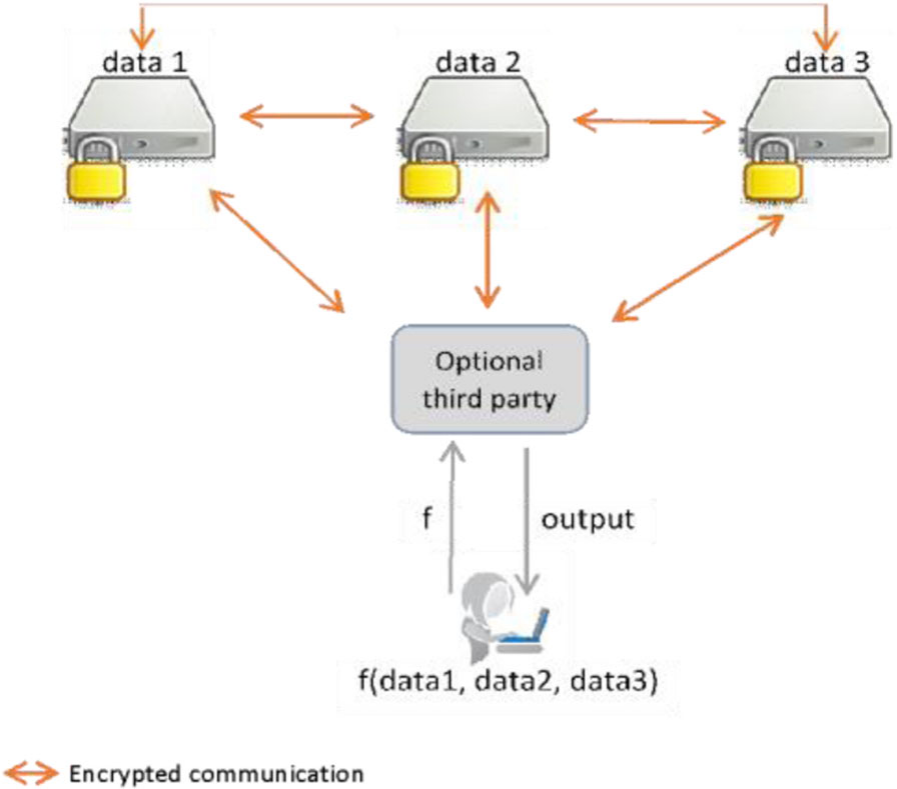
Framework for the secure multiparty computation

Figure 3 illustrates the SMC concept. In the figure, a researcher wants to run a computation *f* over the private inputs of three remote databases (data1, data2, data3) while keeping these inputs private. The different parties are allowed to exchange messages with each other and with the researcher. However, such messages are encrypted so as to prevent the different parties from learning any private information through interaction.

SMC is gaining more popularity in the biomedical domain. SMCs are supported by robust mathematical proofs demonstrating their ability to securely protect privacy and thus proving their ability to support data sharing without fear of privacy abuse. In [66, 67], the authors designed a secure linear regression using homomorphic encryption for a multi-hospital quality improvement study. In [68], a secure genome-wide association study (GWAS) was designed using homomorphic encryption, and in [69], a GWAS protocol was designed using secret sharing. In [70], the authors use garbled circuits to perform metagenomics analysis.

In general, the protocols for secure computation have achieved outstanding results; it has been shown that any function (no matter how complex) can be computed securely. Efficiency however is the major drawback of these computations; they are much more complex than regular protocols (that do not provide any security) [71]. The complexity is driven by the extensive message passing between the involved parties as well as the cryptographic functions employed. Recently, the authors in [72] presented a fast and secure computation for linear regression over distributed data based on secure matrix multiplication. And, the authors in [73] designed another efficient secure multiparty linear regression protocol; their method was based on mathematical results in estimation theory. It remains to be seen whether these methods are generalizable to other estimators.

## Dissemination of findings

Prior work demonstrated that in order to affirm the value of research participation and contribute to public education, it is important to have a mechanism for disseminating research findings to the public. This will keep the community aware of how their participation is facilitating research and improving knowledge in the biomedical field.

The mechanism should also tackle the issue of disseminating individual research findings to specific participants. The recommendations governing the return of individual results are usually driven by the psychological harm that could affect the subjects from knowing a result weighted by the benefits in learning it. As such, recommendations are usually aligned with returning “clinically actionable” results, that is, results that are considered *scientifically valid* and that constitute *valuable information* for the recipient, i.e., results associated with some kind of preventive/cautionary strategy.

For example, a finding of deleterious mutations in the BRCA1 or BRCA2 genes associates diagnosed women with high frequency of developing breast or ovarian cancer. Such valid findings help the participants choose to undergo more screening (yearly mammograms, yearly MRI), frequent clinical breast screenings, or bilateral risk-reducing mastectomy which is known to reduce the risk of cancer up to 95% [74–76].

Another example concerns the incidence of mutations in chromosome 12 in the gene coding for phenylalanine hydroxylase (PAH). The mutation may result in the absence of or a defect in PAH enzyme. Phenylketonuria (PKU) can be prevented if PKU is diagnosed soon after birth; children can be placed on diets low in phenylalanine and the detrimental effects of accumulated phenylalanine are avoided. Such highly valuable information for the recipient might prevent severe mental retardation as a result of PKU.

Other findings might not put the participants at risk of developing a disease but could give them the necessary information to guide some of their life choices; an example is whether the participant is a carrier for albinism.

The American College of Medical Genetics and Genomics (ACMG) published a policy statement in 2013 specifying the mutations that should be sought and reported back to the participants (in the context of clinical sequencing). ACMG updates these recommendations annually.

Although the ACMG recommendations were put forth by experts in the field, these underwent a thorough deliberation process and were reviewed (before publication) by external geneticists; they were criticized for excluding the community from the discussion [77]. In fact, there is a growing push to empower members of the public regarding genetic research in general and regarding the return of individual results to research participants in particular. Empirical studies have shown that the majority of participants would like to learn a broader array of genetic results than what is recommended and that they would like to be given the opportunity to decide on that matter [78]. This however necessitates the design of an educational and dynamic consent process to capture the informed (and fluctuating) choices of participants with regards to returning their interpreted data and to continuously educate participants (refer to the “Regulations” section). Such individual consent coupled with educational material could be provided to participants through a secure online portal system for them to complete at their own pace and as the need arises. This allows consent documents to be tied to real events as they occur in the data life cycle, rather than requiring all consent issues to be defined at the beginning of the study. Thus, for example, as new information is generated that changes a variant’s status from ambiguous to actionable, additional educational programs and consent documents can be created to allow participants to decide if they want to receive information about the variant and/or to allow that information to be transmitted to their physicians.

Another difficult issue at the core of information dissemination is that of interpretation of the genome sequence information. Interpretation requires the storage of additional information in a form that is easily understood by medical doctors (and other caregivers). It also necessitates the continuous updating of this information with any relevant findings.

A table summarizing several characteristics of select genome projects is presented at the end of the manuscript (Table 2). For every project, it indicates the target number of genomes to be sequenced, the number of genomes sequenced to date, the project’s context, the initiation date, the data access model (open versus controlled), the consent process, whether it supports notification (or dissemination) of relevant clinical data, and whether a de-identification mechanism is applied.

**Table 2:** Characteristics of selected genome projects. In *opt-out* consent process, consent is presumed (for clinical data and left-over hospital samples) with an opportunity to opt out. *Opt-out* is usually coupled with *paper-based consent* for individuals who want to volunteer samples at the biobank. In *local access* model, researchers are not allowed to download the data; they can only access it on the data holder’s site. – indicates missing information, *Intra-country* indicates that data is not allowed to leave the country (collaborations should be done through a local researcher)

Projects	Declared target #genomes/exomes	#Genomes sequenced to date	Context	Start date	Data access model	Consent process	Notification of relevant data	De-identification process
Human Longevity	1000,000 WGS	–	Research	2013	Controlled	paper based	Yes	Yes
All of US	1000,000 WGS	0	Research	2017	Multi-tier (open to controlled), based on risk of request	Dynamic consent	Yes	Yes
Korean Genome Project	1000 WGS for 2016 10,000 WGS for 2018 50,000,000 WGS for 2030	1722	Research	2012	Open	–	–	Yes
QGP	300,000 WGS	4000	Research	2013	Controlled (multi-ethics/review boards)	Paper-based (11 simple questions)	Yes	Yes
Estonian Genome Project	–	52,000^a^ samples	Research	2000	Controlled (multi-ethics/review boards	broad paper-based consent	Yes	Yes
Saudi Human Genome Program	100,000 WES	–	Research, diagnostic screening	2013	Controlled	Informed Paper based consent	Yes	Yes
Decode Genetics	300,000 WGS (with imputation)	160,000	Research	1996	Controlled (intra-country)	Opt-out/ paper-based consent	Yes	Yes
The Faroe Genome Project	50,000 WGS	–	Research	2011	Controlled (multi-ethics/review boards)	Informed consent (one for each research project)	No^b^	Yes
Genomics England	100,000	52,065	Research	2015	Controlled (access committee)	Paper-based	Yes	Yes, coupled with local access

^a^The number of biological specimens collected up to date

^b^Upon participation in a research study, subjects may opt to receive notification about different genetic results that may be revealed

## Conclusion

Biomedical sciences have been evolving faster than the societies’ ability to cope with them. On one hand, current technical approaches to privacy are not adequate for modern biomedical data, and on the other hand, privacy laws have not been updated to deal with the special features of genomic data. As a result, common practice for biomedical data sharing is either rule-based or relies on an IRB for data-sharing decisions. These processes lack a clear and quantitative measurement of privacy risks.

Moreover, calls for participants’ empowerment and data ownership are increasing. Data ownership gives the right to individuals to be the guardians of their own data, allowing them to access their data, modify it, set access rules, and modify the rules at will. Informed consent is believed to grant such right of self-determination to the individuals by specifying how they like their data to be accessed (data sharing) and what findings (from their data) they would like to receive back (data dissemination).

However, we cannot talk about participants’ empowerment without talking about culture and education. As mentioned earlier in the paper, the right of self-determination is deeply affected by culture. More studies are needed to understand the role of religion, cultures, and traditions in constructing norms around privacy and self-determination.

On the education front, more effort should be made to (continuously and dynamically) educate the public and inform them about the great benefits arising from sharing their data and the potential risk and damage that could result on the individual and their close relatives should their information be breached.

On another related topic, that of genomic medicine, advancements are needed on many fronts to integrate genetic knowledge into medical practice. On one hand, consent issues regarding dissemination of findings should be resolved, and on the other hand, issues that require development are (i) genetic knowledge representation and the technical limitations of EMR systems, (ii) the lack of genetic training programs for practitioners, and (iii) the difficulty in interpreting genetic results (due to their probabilistic nature and their dependency on phenotypic data).

## Abbreviations

ACMG: American College of Medical Genetics and Genomics
APOE: Apolipoprotein E
CAPI: Computer-Assisted Personal Anterview
DNA: DeoxyriboNucleic Acid
DXA: Dual X-ray Absorptiometry
eMERGE: Electronic Medical Records and Genomics
EMR: Electronic Medical Record
EU: European Union
GINA: Genetic Information Non-discrimination Act
GWAS: Genome-Wide Association Study
HIPAA: Health Information Portability and Accountability Act
IRB: Institutional Review Board
NIH: National Institute of Health
NUgene: North Western University biorepository
NUMC: North Western University Medical Center
PAH: PhenylAlanine Hydroxylase
PKU: PhenylKetonUria
PMI: Precision Medicine Initiative
QGP: Qatar Genome Programme
QSCH: Qatar Council for Healthcare Practitioners
SHA-512: Secure Hash Algorithm
SMC: Secure Multiparty Communication
SNP: Single Nucleotide Polymorphism
SSN: Social Security Number
VGER: Vanderbilt Genome-Electronic Records
WGS: Whole Genome Sequencing

## References

[1] Decode genetics. http://www.decode.com/.

[2] Gulcher J, Stefansson K (1999). An Icelandic saga on a centralized healthcare database and democratic decision making. Nat Biotechnol.

[3] Gudbjartsson DF, Helgason H, Gudjonsson SA, Zink F, Oddson A, Gylfason A (2015). Large-scale whole-genome sequencing of the Icelandic population. Nat Genet.

[4] Genome England. http://genomicsengland.co.uk.

[5] Human Longevity. http://www.humanlongevity.com/.

[6] Precision Medicine Initiative. http://www.nih.gov/precisionmedicine/.

[7] Cyranoski D (2016). China embraces precision medicine on a massive scale. Nat News.

[8] Korean Reference Genome Project. http://152.99.75.168/KRGDB/menuPages/intro.jsp.

[9] Abu-Elmagd M, Assidi M, Schulten H-J, Dallol A, Pushparaj PN, Ahmed F (2015). Individualized medicine enabled by genomics in Saudi Arabia. BMC Merd Genomics.

[10] Qatar BioBank. http://www.qatarbiobank.org.qa/media-center/event-detail?item=33&backArt=29.

[11] The DNA law. Kuwait Times. 2016. http://news.kuwaittimes.net/website/the-dna-law/. Accessed 26 Feb 2017.

[12] Nyholt DR, Yu C-E, Visscher PM (2009). On Jim Watson’s APOE status: genetic information is hard to hide. Eur J Hum Genet.

[13] El Emam K (2011). Methods for the de-identification of electronic health records for genomic research. Genome Med.

[14] Gelfand A (2012). Privacy and biomedical research: building a trust infrastructure.

[15] Cassa CA, Schmidt B, Kohane IS, Mandl KD. My sister’s keeper?: genomic research and the identifiability of siblings. BMC Med Genomics. 2008;1. 10.1186/1755-8794-1-32. 10.1186/1755-8794-1-32 PMC2503988 18655711

[16] Naveed M, Ayday E, Clayton EW, Fellay J, Gunter CA, Hubaux J-P (2015). Privacy in the genomic era. ACM Comput Surv CSUR.

[17] Dankar FK, Al-Ali R (2016). Building of a large scale de-identified biomedical database in Qatar—principles and challenges. Qatar Found Annu Res Conf Proc.

[18] McCarty CA, Chisholm RL, Chute CG, Kullo IJ, Jarvik GP, Larson EB, et al. The eMERGE network: a consortium of biorepositories linked to electronic medical records data for conducting genomic studies. BMC Med Genomics. 2011;4:13. 10.1186/1755-8794-4-13 PMC3038887 21269473

[19] Roden DM, Pulley JM, Basford MA, Bernard GR, Clayton EW, Balser JR (2008). Development of a large-scale de-identified DNA biobank to enable personalized medicine. Clin Pharmacol Ther.

[20] All About The Human Genome Project (HGP). National Human Genome Research Institute (NHGRI). https://www.genome.gov/10001772/All-About-The%2D-Human-Genome-Project-HGP. Accessed 26 Feb 2017.

[21] McGuire AL, Oliver JM, Slashinski MJ, Graves JL, Wang T, Kelly PA (2011). To share or not to share: a randomized trial of consent for data sharing in genome research. Genet Med..

[22] McGuire AL, Hamilton JA, Lunstroth R, McCullough LB, Goldman A (2008). DNA data sharing: research participants’ perspectives. Genet Med..

[23] Oliver JM, Slashinski MJ, Wang T, Kelly PA, Hilsenbeck SG, McGuire AL (2011). Balancing the risks and benefits of genomic data sharing: genome research participants’ perspectives. Public Health Genomics..

[24] Abokhodair N, Vieweg S (2016). Privacy & social media in the context of the Arab Gulf. Proceedings of the 2016 ACM Conference on Designing Interactive Systems.

[25] Hubbard T. HPC infrastructure at King’s College London and Genomics England. In: Farr-ADRN-MB eInfrastructure Workshop. 2015.

[26] Leitsalu L, Metspalu A, Ginsburg GS, Willard HF (2017). Chapter 8—from biobanking to precision medicine: the Estonian experience. Genomic and precision medicine.

[27] Abouelhoda M. The informatics side of the Saudi human genome project. In: GenoME. 2016.

[28] Ayday E, Cristofaro ED, Hubaux JP, Tsudik G. The Chills and Thrills of Whole Genome Sequencing. IEEE Computer Magazine; 2013.

[29] Wang S, Jiang X, Singh S, Marmor R, Bonomi L, Fox D (2017). Genome privacy: challenges, technical approaches to mitigate risk, and ethical considerations in the United States. Ann N Y Acad Sci.

[30] ExAC Browser. http://exac.broadinstitute.org/about. Accessed 6 Mar 2018.

[31] Check Hayden E (2013). Privacy protections: the genome hacker. Nature.

[32] Gymrek M, McGuire AL, Golan D, Halperin E, Erlich Y (2013). Identifying personal genomes by surname inference. Science.

[33] Sweeney L, Abu A, Winn J (2013). Identifying participants in the personal genome project by name.

[34] Homer N, Szelinger S, Redman M, Duggan D, Tembe W, Muehling J (2008). Resolving individuals contributing trace amounts of DNA to highly complex mixtures using high-density SNP genotyping microarrays. PLoS Genet.

[35] Malin B, Cassa C, Kantarcioglu M. A survey of challenges and solutions for privacy in clinical genomics data mining. In: Bonchi F, Ferrari E, editors. Privacy-Preserving Knowledge Discovery. New York: Chapman & Hall/CRC Press; 2011.

[36] Federal policy for the protection of human subjects (‘Common Rule’). http://www.hhs.gov/ohrp/humansubjects/commonrule/.

[37] Sweeney L. Data sharing under HIPAA: 12 years later. In: Workshop on the HIPAA Privacy Rule’s de-identification standard. 2010.

[38] U.S. Department of Health & Human Services. http://www.hhs.gov/. Accessed 22 Sept 2015.

[39] European Data Protection Directive. https://ico.org.uk/media/about-the-ico/documents/1042349/review-of-eu-dp-directive.pdf.

[40] Murphy SN, Weber G, Mendis M, Gainer V, Chueh HC, Churchill S (2010). Serving the enterprise and beyond with informatics for integrating biology and the bedside (i2b2). J Am Med Inform Assoc JAMIA.

[41] Wolf LE, Walden JF, Lo B (2005). Human subjects issues and IRB review in practice-based research. Ann Fam Med.

[42] Graham DG, Spano MS, Manning B (2007). The IRB challenge for practice-based research: strategies of the American Academy of Family Physicians National Research Network (AAFP NRN). J Am Board Fam Med.

[43] He S, Narus SP, Facelli JC, Lau LM, Botkin JR, Hurdle JF (2014). A domain analysis model for eIRB systems: addressing the weak link in clinical research informatics. J Biomed Inform.

[44] Silberman G, Kahn KL (2011). Burdens on research imposed by institutional review boards: the state of the evidence and its implications for regulatory reform. Milbank Q.

[45] Appelbaum PS, Waldman CR, Fyer A, Klitzman R, Parens E, Martinez J (2013). Informed consent for return of incidental findings in genomic research. Genet Med.

[46] Ohno-Machado L, Bafna V, Boxwala AA, Chapman BE, Chapman WW, Chaudhuri K, et al. iDASH: integrating data for analysis, anonymization, and sharing. J Am Med Inform Assoc. 2011;19(2):196-201. 10.1136/amiajnl-2011-000538 PMC3277627 22081224

[47] Whitley EA, Kanellopoulou N, Kaye J (2012). Consent and research governance in biobanks: evidence from focus groups with medical researchers. Public Health Genomics.

[48] Steinsbekk KS, Myskja BK are, Solberg B. Broad consent versus dynamic consent in biobank research: is passive participation an ethical problem? Eur J Hum Genet 2013;21:897–902. 10.1038/ejhg.2012.282 PMC3746258 23299918

[49] Goetz T. 23andMe will decode your DNA for $1,000: welcome to the age of genomics. Wired Mag. 2007. http://davehakes.com/weblog/wp-content/uploads/2007/11/11-17-07_wired_welcome_to_the_age_of_genomics.pdf. Accessed 26 Feb 2017.

[50] McGuire AL, Beskow LM (2010). Informed consent in genomics and genetic research. Annu Rev Genomics Hum Genet.

[51] Grady, C. Enduring and emerging challenges of informed consent. New Engl J Med. 2015;372(9):855-62. 10.1056/NEJMra1411250 25714163

[52] Dankar FK, Badji R (2017). A risk-based framework for biomedical data sharing. J Biomed Inform.

[53] Adams A. The implications of users’ multimedia privacy perceptions on communication and information privacy policies. In: Proceedings of Telecommunications Policy Research Conference. 1999. http://www.researchgate.net/profile/Anne_Adams4/publication/228641284_The_Implications_of_Users’_Multimedia_Privacy_Perceptions_on_Communication_and_Information_Privacy_Policies/links/0046352a751ad9c417000000.pdf. Accessed 14 Jun 2015.

[54] Lederer S, Dey AK, Mankoff J (2002). A conceptual model and a metaphor of everyday privacy in ubiquitous.

[55] Lessig L (1999). Architecture of privacy. The Vand J Ent Pr.

[56] Barker K, Askari M, Banerjee M, Ghazinour K, Mackas B, Majedi M, et al. A data privacy taxonomy. In: Dataspace: the final frontier. Springer; 2009. p. 42–54. http://link.springer.com/chapter/10.1007/978-3-642-02843-4_7. Accessed 7 Jul 2015.

[57] Banerjee M, Adl RK, Wu L, Barker K. Quantifying privacy violations. In: Secure data management. Springer; 2011. p. 1–17. http://link.springer.com/chapter/10.1007/978-3-642-23556-6_1. Accessed 7 Jul 2015.

[58] El Emam K, Dankar FK, Vaillancourt R, Roffey T, Lysyk M (2009). Evaluating the risk of re-identification of patients from hospital prescription records. Can J Hosp Pharm.

[59] El Emam K (2010). Risk-based de-identification of health data. IEEE Secur Priv.

[60] El Emam K, Jonker E, Fineberg A. The case for de-identifying personal health information. Soc Sci Res Netw. 2011. http://papers.ssrn.com/sol3/papers.cfm?abstract_id=1744038. Accessed 18 Sep 2012.

[61] Dankar FK, El Emam K, Neisa A, Roffey T (2012). Estimating the re-identification risk of clinical data sets. BMC Med Inform Decis Mak.

[62] El Emam K, Dankar FK, Neisa A, Jonker E (2013). Evaluating the risk of patient re-identification from adverse drug event reports. BMC Med Inform Decis Mak..

[63] Dankar FK, Al-Ali R (2015). A theoretical multi-level privacy protection framework for biomedical data warehouses. Procedia Comput Sci.

[64] Cogo VV, Bessani A, Couto FM, Verissimo P (2015). A high-throughput method to detect privacy-sensitive human genomic data. Proceedings of the 14th ACM Workshop on Privacy in the Electronic Society.

[65] Dyke SO, Dove ES, Knoppers BM. Sharing health-related data: a privacy test? Npj Genomic Med 2016;1:16024. 10.1038/npjgenmed.2016.24 PMC5158304 27990299

[66] Dankar F, Brien R, Adams C, Matwin S. Secure multi-party linear regression. In: EDBT/ICDT workshops. 2014. p. 406–414. http://ceur-ws.org/Vol-1133/paper-68.pdf. Accessed 14 Jan 2015.

[67] Dankar F (2015). Privacy preserving linear regression on distributed databases. Trans Data Priv.

[68] Ugwuoke C, Erkin Z, Lagendijk I. A Privacy-Preserving GWAS Computation with Homomorphic Encryption. 37th WIC Symposium on Information Theory in the Benelux/6th WIC/IEEE SP Symposium on Information Theory and Signal Processing in the Benelux, Louvain, Belgium. 2016. p. 166-73. https://pure.tudelft.nl/portal/files/11312239/11312198.pdf.

[69] Kamm L, Bogdanov D, Laur S, Vilo J (2013). A new way to protect privacy in large-scale genome-wide association studies. Bioinformatics.

[70] Wagner J, Paulson JN, Wang X, Bhattacharjee B, Corrada Bravo H. Privacy-preserving microbiome analysis using secure computation. Bioinformatics. 2016;32(12):1873-9. 10.1093/bioinformatics/btw073 PMC4908319 26873931

[71] Lindell Y, Pinkas B (2009). Secure multiparty computation for privacy-preserving data mining. J Priv Confidentiality.

[72] de CM, Dowsley R, Nascimento ACA, Newman SC (2015). Fast, privacy preserving linear regression over distributed datasets based on pre-distributed data. Proceedings of the 8th ACM Workshop on Artificial Intelligence and Security.

[73] Dankar FK, Boughorbel S, Badji R. Using robust estimation theory to design efficient secure multiparty linear regression. In: Proceedings of the 2016 Joint EDBT/ICDT Workshops. 2016. http://ceur-ws.org/Vol-1558/paper33.pdf. Accessed 9 Sept 2016.

[74] Rebbeck TR, Friebel T, Lynch HT, Neuhausen SL, van’t Veer L, Garber JE (2004). Bilateral prophylactic mastectomy reduces breast cancer risk in BRCA1 and BRCA2 mutation carriers: the PROSE study group. J Clin Oncol.

[75] Domchek SM, Friebel TM, Singer CF, Evans DG, Lynch HT, Isaacs C (2010). Association of risk-reducing surgery in BRCA1 or BRCA2 mutation carriers with cancer risk and mortality. JAMA.

[76] Hartmann LC, Schaid DJ, Woods JE, Crotty TP, Myers JL, Arnold PG (1999). Efficacy of bilateral prophylactic mastectomy in women with a family history of breast cancer. N Engl J Med.

[77] Terry SF (2013). Don’t just invite us to the table: authentic community engagement. Genet Test Mol Biomark.

[78] Dresser R (2014). Public preferences and the challenge to genetic research policy. J Law Biosci.

